# Cancer Informatics: Novel Methods and Applications of Artificial Intelligence in Cancer Care Delivery

**DOI:** 10.1055/s-0044-1800727

**Published:** 2025-04-08

**Authors:** Sanjay Aneja, Ravi B. Parikh

**Affiliations:** 1 Yale Cancer Center, New Haven, CT, USA; 2 Department of Therapeutic Radiology, Yale School of Medicine, New Haven, CT, USA; 3 Department of Bioinformatics and Data Science, Yale School of Medicine, New Haven, CT, USA; 4 Abramson Cancer Center, Philadelphia, PA; 5 Department of Medicine, Philadelphia, PA; 6 Department of Medical Ethics and Health Policy, Philadelphia, PA

**Keywords:** Neoplasms, informatics, health information technology, clinical implementation

## Abstract

**Objectives** : To summarize significant research contributions on cancer informatics published in 2023, an extensive search using PubMed/MEDLINE was conducted to identify the scientific contributions published in 2023 that address topics in cancer. The selection process comprised three steps: (i) ten candidate best papers were first selected by the two section editors, (ii) external reviewers from internationally renowned research teams reviewed each candidate best paper, and (iii) the final selection of three best papers was conducted by the editorial board of the Yearbook.

**Results** : The two selected papers demonstrate advances in the clinical implementation of cancer informatics methodologies. Both studies highlight translation of informatics methodologies to improve cancer outcomes.

**Conclusions** : Cancer informatics is a maturing subfield of bioinformatics. As novel methodologies continue to emerge, further emphasis will be placed on rigorous clinical validation and real-world scalability of such solutions to positively impact patient outcomes.

## 1. Introduction

Cancer informatics (CI) is a broad field with several fundamental goals: 1) organizing data in ways that are comprehensible and meaningful to clinicians, researchers, and patients; 2) using data to advance the treatment of cancer; and 3) manipulating data to yield new insights. In this edition of the Cancer Informatics section, the focus has shifted from the increasing important area of clinical implementation. Whereas previous efforts in cancer informatics have been largely focused on the development of new methodologies to model cancer data, recently there has been an emphasis on translating such methodologies within clinical practice. Both papers selected for the 2024 describe informatics tools that either have been rigorously validated in the context of a randomized clinical trial or leverage data that is relatively scalable across clinical settings.

## 2. Paper Selection Method

One electronic database was searched: PubMed/MEDLINE. The search was performed in January 2024 to identify peer-reviewed journal articles published in 2023, in the English language, related to cancer informatics research. The following search was implemented:

This is identical to the search for papers in 2023 except that we also added terms related to artificial intelligence and machine learning. This search yielded 6,366 results. Next, we excluded review articles, resulting in 1,826 articles for first-pass review. The titles of these articles were blindly screened for relevance, resulting in 270 articles that were reviewed in further depth. The abstract of each of the 270 was blindly reviewed and assigned as potential candidate (n=35) and non-candidate (n=235). Given that the number of potential candidates exceeded ten, these articles were further evaluated to select twelve final candidates.

In accordance with the IMIA Yearbook selection process, the 12 candidate best papers were evaluated by the two section editors, senior editors, and by additional external reviewers (at least four reviewers per paper) [ 1 ]. The geographic distribution of the reviewers was: eight United States, two United Kingdom, one France, and one Denmark. Two papers were finally selected as best papers ( Table 1 ). A content summary of the selected best papers can be found in the appendix of this synopsis.

**Table 1.** Selection of best papers for the 2024 IMIA Yearbook of Medical Informatics for the section Clinical Research Informatics. The articles are listed in alphabetical order of the first author's surname.

Section Cancer Informatics
Manz C et al. Long-term Effect of Machine Learning-Triggered Behavioral Nudges on Serious Illness Conversations and End-of-Life Outcomes Among Patients With Cancer: A Randomized Clinical Trial. JAMA Oncology. 2023 Mar 1;9(3):414-418. doi: 10.1001/jamaoncol.2022.6303. Placido D et al. A Deep Learning Algorithm To Predict Risk Of Pancreatic Cancer From Disease Trajectories. Nature Medicine. 2023 May;29(5):1113-1122. doi: 10.1038/s41591-023-02332-5.

## 3. Outlook

The 12 candidate best papers for 2024 illustrate recent efforts towards data-driven research and innovation and exemplify a diversity of different data streams across cancer informatics. Studies analyzed data ranging from administrative codes, structured electronic health record data, diagnostic images, and pathology imaging. The two selected best papers cover two different areas of the oncology care spectrum, namely cancer screening and end-of-life care. The diversity in papers highlights the maturation of cancer informatics as a subfield and the wealth of opportunities to leverage oncology data to personalize care.

Manz et al. [ 2 ] conduct a randomized clinical trial to evaluate whether a machine learning algorithm which predicts mortality could be combined with behavioral interventions to improve end-of-life care for patients with advanced cancer. In a trial that spanned nine clinical practices, the authors found that clinicians exposed to the machine learning-driven behavioral intervention were three times more likely to have early goals of care conversations and less likely to use end-of-life systemic therapy.

Placido et al. [ 3 ] developed longitudinal machine learning methods based on diagnosis codes in routine administrative data, trained and validated on 6 million patients (24,000 pancreatic cancer cases) in Denmark and 3 million patients (3,900 cases) in the United States (US Veterans Health Administration). Their models predicted pancreatic cancer occurrence within incremental time windows. For the Danish cohort, the area under the receiver operating characteristic (AUROC) curve was 0.88, with an estimated relative risk of 59 for 1,000 highest-risk patients older than age 50 years. For the US Veteran cohort, the best-performing AUCROC=0.78.

The other 10 candidate papers cover a variety of different aspects of cancer informatics and we outline those papers below. These papers highlight clinical model infrastructure in cancer research, validation of machine learning models for cancer, artificial intelligence (AI) models for diagnosis, and a special paper on federated learning. With new tools particularly in AI being developed, the cancer informatics field continues to be very exciting.

### 

#### 

##### Building model infrastructure

Integrative Imaging Informatics for Cancer Research: Workflow Automation for Neuro-Oncology (3CR-WANO). Journal of Clinical Oncology Clinical Cancer Informatics, 2023 [ 4 ].

Precision Oncology Core Data Model to Support Clinical Genomics Decision Making. Journal of Clinical Oncology Clinical Cancer Informatics, 2023 [ 5 ].

The Childhood Cancer Data Initiative: Using the Power of Data to Learn from and Improve Outcomes for Every Child and Young Adult with Pediatric Cancer, Journal of Clinical Oncology, 2023 [ 6 ].

##### Prospective validation of machine learning models

Prospective implementation of AI-assisted screen reading to improve early detection of breast cancer. Nature Medicine, 2023 [ 7 ].

Screening for extranodal extension in HPV-associated oropharyngeal carcinoma: Evaluation of a CT-based deep learning algorithm in patient data from a multicenter, randomized de-escalation trial. Lancet Digital Health, 2023 [ 8 ].

##### AI models focused on diagnosis of malignancy

Artificial-intelligence-based molecular classification of diffuse gliomas using rapid, label-free optimal imaging. Nature Medicine, 2023 [ 9 ].

A reinforcement learning model for AI-based decision support in skin cancer. Nature Medicine, 2023 [ 10 ].

##### Federated Learning

Federated Learning for Predicting Histological Response to Neoadjuvant Chemotherapy in Triple-Negative Breast Cancer. Nature Medicine, 2023 [ 11 ].

## 4. Conclusion

The best papers for cancer informatics present promising frameworks and applications for novel informatics infrastructure and artificial intelligence applications in cancer care. We were pleased to see an emphasis on prospective applications of artificial intelligence beginning to be represented.

## Notes

((“Neoplasms”[Mesh] OR “chemotherapy”) AND (“Informatics”[Mesh] OR “cancer informatics” OR “ontologies” OR “machine learning” OR “artificial intelligence”) AND (hasabstract[text] AND (“2023/01/01”[PDAT] : “2023/12/31”[PDAT]) AND English[lang])) NOT (“Radiotherapy Planning, Computer-Assisted”[Mesh]) NOT (“Radiotherapy, Computer-Assisted”[Mesh])
